# Massive intracranial hemorrhage caused by intraventricular meningioma: case report

**DOI:** 10.1186/s12883-021-02056-4

**Published:** 2021-01-16

**Authors:** Koichiro Sumi, Takeshi Suma, Reona Yoshida, Ryuta Kajimoto, Masato Kobayashi, Takamichi Katsuhara, Koki Hirayama, Xiaoyan Tang, Naoki Otani, Atsuo Yoshino

**Affiliations:** 1 Department of Neurological Surgery, Nihon University School of Medicine, 30-1 Oyaguchi-Kamimachi, Itabashi-ku, Tokyo, 173-8610 Japan; 2 Department of Pathology and Microbiology, Nihon University School of Medicine, Tokyo, Japan

**Keywords:** Meningioma, Intraventricular tumor, Intraventricular hemorrhage, Fibrous meningioma

## Abstract

**Background:**

Meningiomas are the most common benign intracranial tumors, and commonly comprise high-vascularizing but slow-growing tumors. On the other hand, meningiomas arising from the ventricular system are of rare occurrence, and spontaneous hemorrhage is an infrequent event.

**Case presentation:**

We describe here the rare clinical manifestations of a 28-year-old female with acute intracranial hemorrhage located in the trigone of the lateral ventricle who was initially thought to have suffered an acute cerebrovascular accident, but was subsequently confirmed to have a benign intraventricular meningioma. To clarify the clinical features of such a rare course of meningioma, we also present a short literature review of acute intracranial hemorrhage caused by intraventricular meningioma.

**Conclusions:**

Ventricular meningioma presenting with hemorrhage such as acute stroke is a rare event, but recognition of such a pathogenesis is important. Although further accumulation of clinical data is needed, we suggest that early surgery should be undertaken in patients with lateral ventricular meningioma, even if it is not so large or asymptomatic.

## Background

Meningiomas are the most common benign intracranial tumors, accounting for 19 % of all primary intracranial neoplasms, and occur more frequently over the age of 30 years and predominantly in females (approximately 2:1) [1–7]. They commonly comprise high-vascularizing but slow-growing tumors [5, 7]. However, spontaneous hemorrhage, often subarachnoid, subdural, intratumoral, or intracerebral hemorrhage (ICH), is an infrequent event (0.5 % − 2.4 %) [1, 2, 4–11]. An onset mimicking acute stroke such as cerebrovascular accident is even more rare [3, 4, 9, 10].

We describe here the rare clinical manifestations of a 28-year-old female with an apoplectiform onset of intracranial hemorrhage who was initially thought to have suffered an acute cerebrovascular accident, but was subsequently confirmed to have a benign intraventricular meningioma in the trigone. To clarify the clinical features of such a rare course of meningioma, we also present a short literature review of acute intracranial hemorrhage caused by intraventricular meningioma.

### Case presentation

**Fig. 1:**
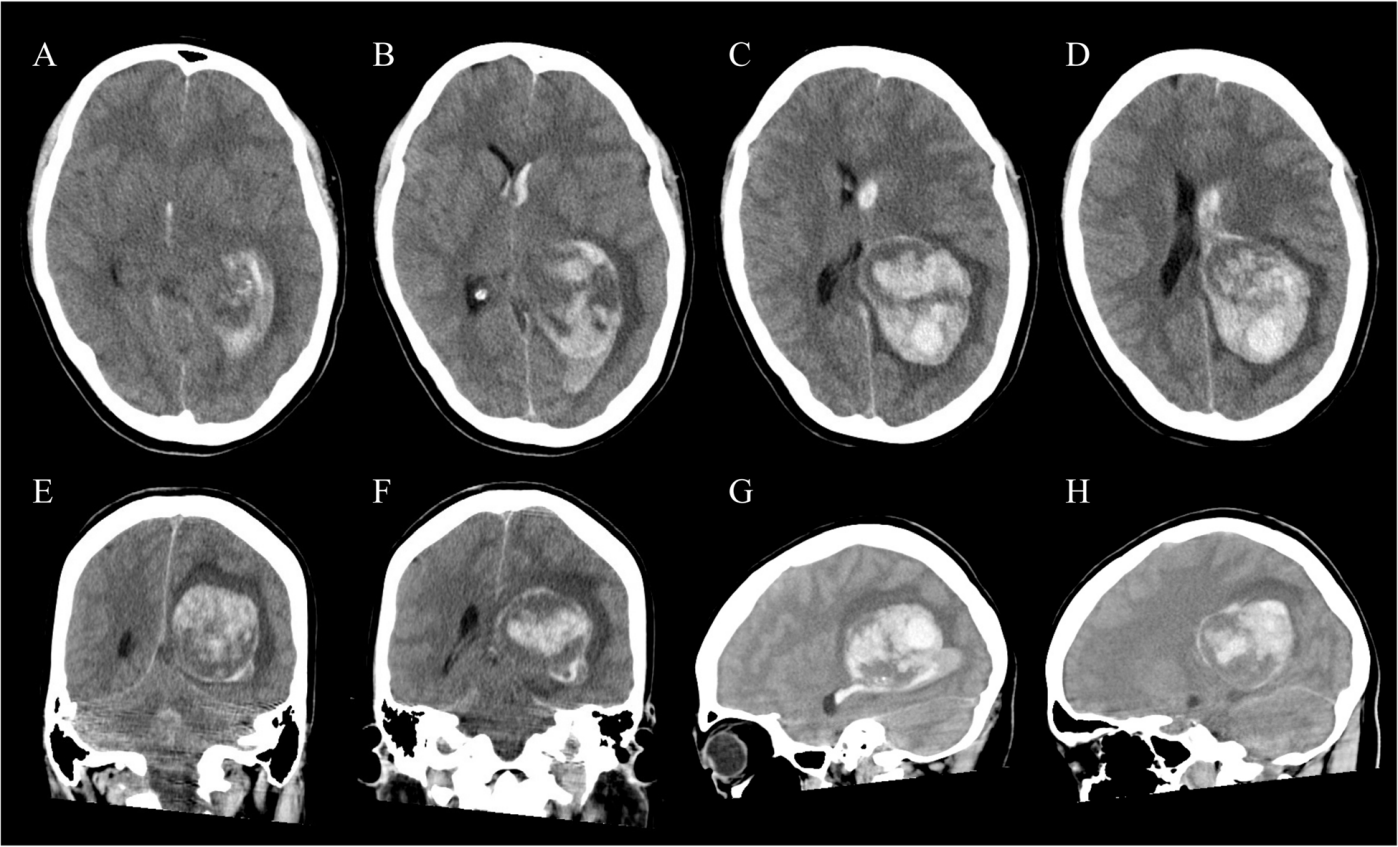
Non-contrast axial (**a**-**d**), coronal (**e**, **f**), and sagittal (**g**, **h**) CT scans on admission revealing a large heterogeneous mass lesion in the trigone of the left lateral ventricle with intraventricular hemorrhage extending to the right lateral ventricle

A 28-year-old female was referred to our hospital with sudden severe headache and vomiting. She fell into a coma during transportation, and was initially treated in the intensive care unit. Prior to this event, she had been in good health except that she often experienced migraine-like headaches. On admission, she was assessed as Glasgow Coma Scale 4 (E1V1M2), and her blood pressure was 125/84 mmHg, heart rate was 67/min (regular in rhythm), respiratory rate was 16/min, body temperature was 36.2^o^C, and routine laboratory blood tests showed no remarkable abnormalities. Her pupils were 2.5-mm prompt when reacting to direct light on the right side and 3.0-mm sluggish when reacting to direct light on the left side. Non-contrast computed tomography (CT) of her head revealed a large heterogeneous-dense space-occupying lesion in the trigone of the left lateral ventricle with midline shift to the right. The oval-shaped lesion was approximately 6.5 × 5.0 × 5.0 cm in size with intraventricular hemorrhage (IVH), which extended forwards into the third and fourth ventricles (Fig. 1). CT angiography showed no vascular abnormality such as aneurysm, vascular malformation, or tumor stain. The patient was thus a relatively young female with no symptoms until onset who had developed a massive intracranial hemorrhage, which made it difficult initially to consider hemorrhage due to meningioma.

**Fig. 2:**
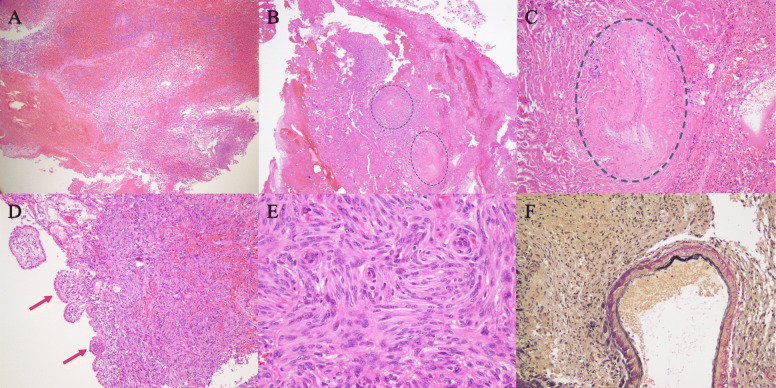
Histological findings of the 1st surgical specimen. Massive hemorrhage was observed within the tumor (**a**: HE stain, x 2). Two closed arteries circled by the blue dotted line were identified in the peri-tumoral abundant collagen regions (**b**: HE stain, x 2). High magnification of one of the closed arteries (**c**: HE stain, x 40). The tumor involved the choroid plexus (**d**: HE stain, x 10). This tumor is consistent with fibrous type meningioma composed of spindle-shaped tumor cells with a fascicular or storiform arrangement (**e**: HE stain, x 20). In addition, dilated abnormal vessels can be seen, of which the walls are thin and the internal elastic membrane appears fragile (**f**: Elastic-van Gieson stain, x 20)

**Fig. 3:**
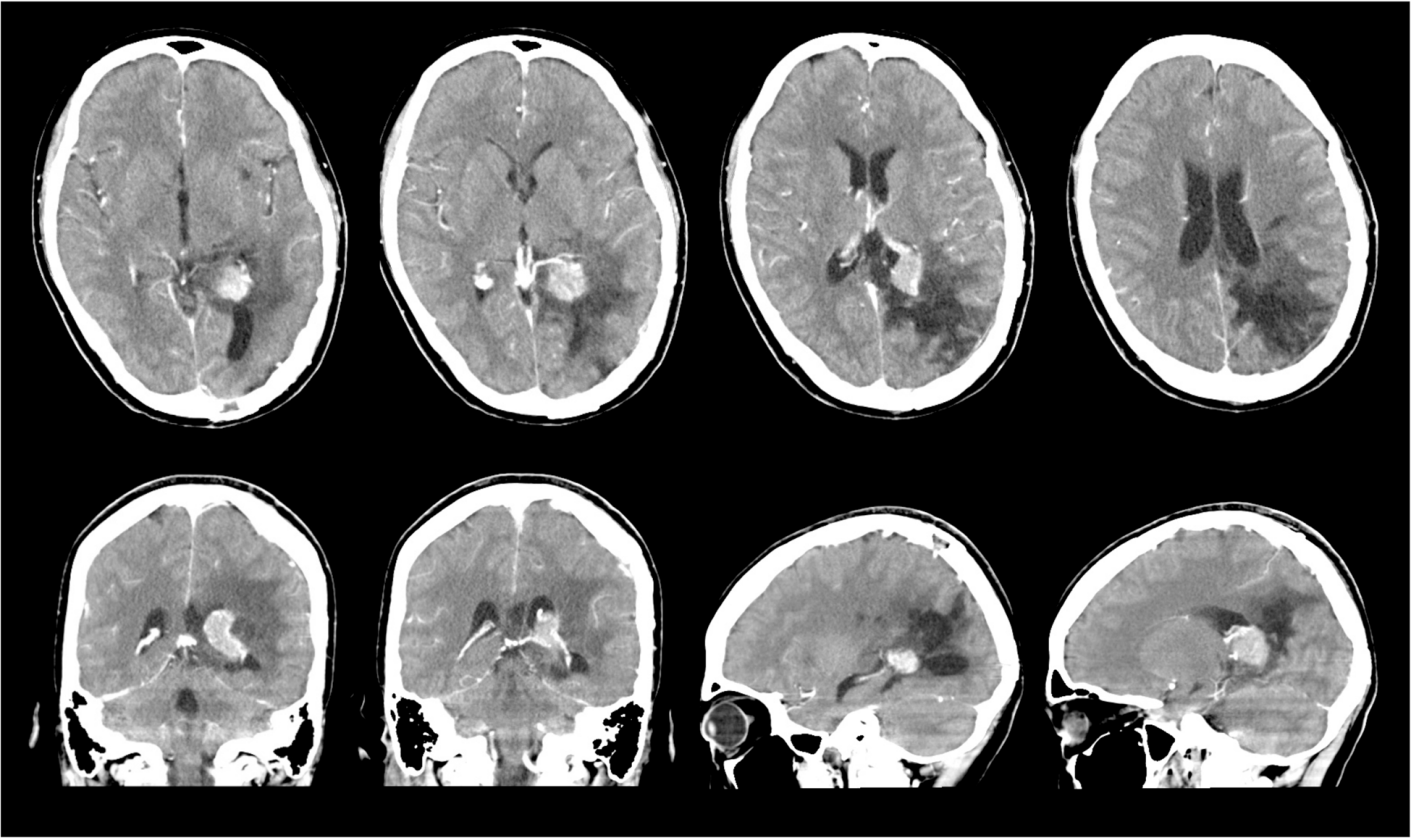
Postcontrast CT scan on day 38 after first surgery showing an enhanced small residual tumor in the trigone of the left lateral ventricle and enlarged feeding artery

Our first impression derived from these findings was acute intracranial hemorrhage in the left ventricular trigone, although the cause of the hemorrhage was unclear. Her clinical status was so bad that urgent extraction of the hematoma was performed in order to save her life. A massive hematoma was removed and the enlarged left lateral ventricle was opened through the occipital pole cortex via a left occipitoparietal craniotomy. The hematoma consisted of one part that could be easily evacuated and another part, located mainly on the deep side, that was solid and bled easily. The surgery was terminated after confirming that a large amount of the hematoma had been debulked, leaving a portion in which hemostasis was difficult. Histological examinations of the solid part revealed a meningioma with massive hemorrhage within the tumor and many irregular vessels, including softened thin-walled vessels, closed vessels, etc., at the peripheral area of the tumor (Fig. 2). Closed arteries with organized reaction suggested a possibility of old hemorrhage. Spindle-shaped tumor cells were arranged in fascicular or storiform structures, and a few psammoma bodies were observed. Nuclear atypia or mitosis was rarely seen, and the Ki-67 proliferative index was less than 3 %. The tumor also extended to the choroid plexus, suggesting its derivation. The diagnosis of the 1st surgical specimen was fibrous meningioma, WHO grade 1. The intracranial hematoma was therefore thought to be due to intratumoral hemorrhage in a left lateral ventricular trigone meningioma. Postoperatively, the patient recovered well, but was diagnosed with right homonymous hemianopsia, right hemiparesis, and right abducens nerve palsy. Cerebral angiography was performed 5 days after the first operation and did not show any abnormality such as aneurysm, vascular malformation, or tumor satin. However, follow-up head CT demonstrated a residual tumor in the trigone of the left lateral ventricle (Fig. 3).

**Fig. 4:**
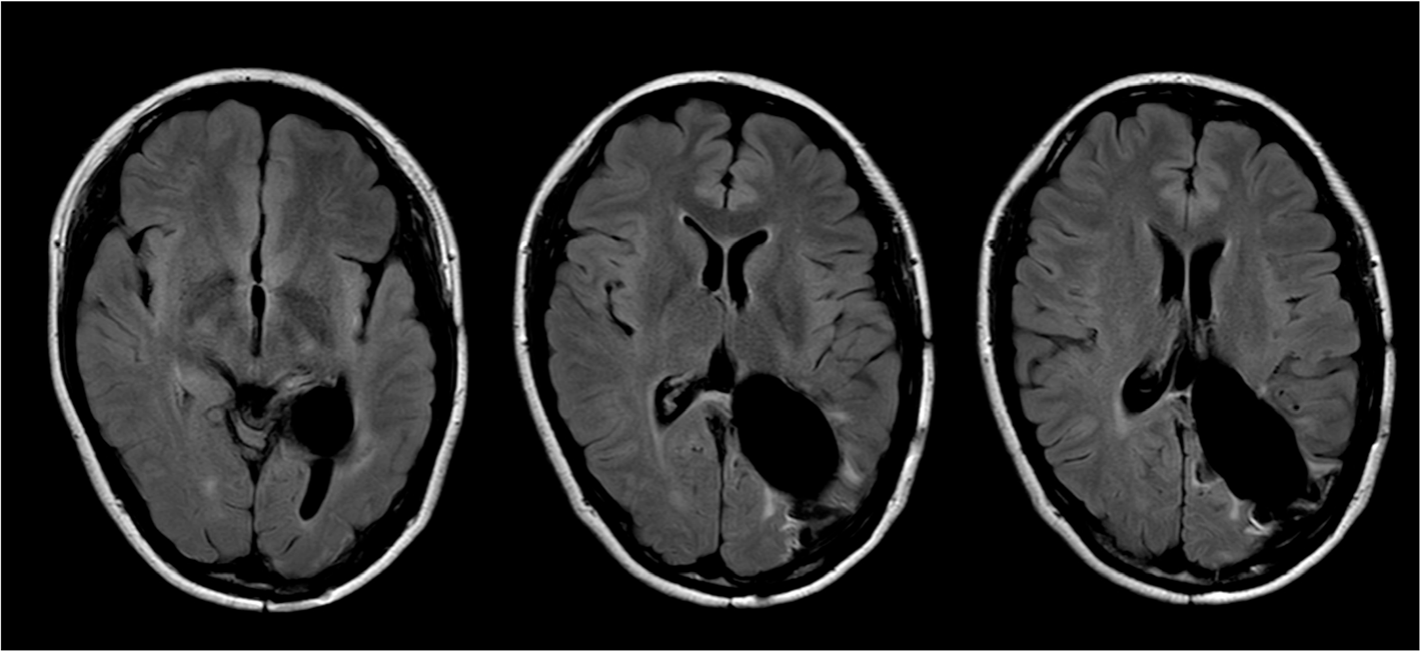
Postoperative axial fluid-attenuated inversion recovery MRI at 15 months after 2nd surgery showing total removal of the meningioma in the trigone of the left lateral ventricle

At 42 days after admission to our institution, 2nd surgery was performed to remove the residual tumor using the same approach as in the 1st surgery. The tumor was found to be relatively soft and well-demarcated. It arose from the choroid plexus, with arterial blood supplied from the medial and posterior lateral choroidal arteries, and was removed completely. Microscopic examinations of the 2nd surgical specimen yielded a diagnosis of fibroblastic meningioma with a Ki-67 proliferative index of l − 2 %. Abundant hemosiderin deposits, indicating prior hemorrhage, were visible within the tumor. The patient’s 2nd postoperative course was uneventful, and no residual intraventricular tumor was found on her follow-up imaging (Fig. 4). She was discharged from our hospital after the 30th post-2nd-surgery day. She was seen to be in a good state at the 15-month follow-up, demonstrating recovery from her right hemiparesis and right abducens nerve palsy, although her hemianopsia was not resolved.

## Discussion and conclusions

Meningiomas arising from the ventricular system are rare in their occurrence, accounting for 0.5–5 % of all intracranial meningiomas, and the most common region is within the trigone of the lateral ventricle [3, 4, 12–21]. Intraventricular meningiomas are thought to arise from either the arachnoid tissue of the choroid plexus or the outer membrane of vessels in the lateral ventricles [3, 4, 21]. The clinical manifestations of intraventricular meningiomas, including within the trigone of the lateral ventricle, are usually asymptomatic until the tumors have grown very large, since the ventricular cavity may provide the tumor room to grow to a certain size before causing issues. As the size of the tumor increases, it presents with a variety of symptoms associated with an elevated intracranial pressure, obstruction of cerebrospinal fluid flow, and pressure on the surrounding brain parenchyma [3, 6, 16, 18, 19, 21]. Intraventricular meningiomas presenting with acute stroke-like intracranial hemorrhage are extremely rare [4, 19]. To the best of our knowledge, after undertaking a careful search of the literature, we identified 13 cases of such hemorrhagic meningioma in the lateral ventricle (including our own case) as summarized in Table 1 [3, 4, 6, 9, 10, 12, 14, 19, 21–23]. Based on the available data collected, the patient age of onset ranged from 14 to 69 years (mean: 44.8 years), the gender ratio was 9:4 with a female predominance, the left-right ratio was 8:5 with a left side predominance, most tumors were located in the trigone, and fibrous meningioma (7 of 13 cases) was the most frequent histological subtype. These features were not different from those of common lateral ventricular meningiomas [4, 10, 19]. Nundkumar et al. and Minderman et al. have reported their experience with stereotactic radiotherapy for 2 and 5 intraventricular trigonal meningiomas (ITM) (not acute stroke-like onset), respectively; and, interestingly, all patients were female and all had tumor of the left ventricle [24, 25]. On the other hand, when compared to a large review of intracranial meningioma bleeding at all intracranial locations undertaken by Bosnjak et al., the patients’ mean age of onset of intraventricular meningioma hemorrhage was about 10 years younger with a female predominance (a review by Bosnjak et al. revealed no gender differences in all intracranial hemorrhagic meningiomas) [5]. The overall mortality rate of hemorrhagic onset of intraventricular meningiomas in our collected cases was about 31 %. In contrast, the mortality rate in the Bosnjak et al. study was 21 % including many pre-CT era cases [5]. In other words, they included cases that were not investigated at the modern medical level. Therefore, the mortality rate of intracranial hemorrhage due to intraventricular meningioma appeared to be high among all sites of meningiomas.

**Table 1:** Summary of the reported cases presenting with acute intracranial hemorrhage associated with lateral intraventricular meningioma

Authors	Year	Age	Sex	Side	Tumor location	Bleeding type	Size at onset	Histology	Consciousness at onset	Outcome	
Askenasy & Behmoaram	1960	34	F	L	trigone	IVH (SAH)	large	psammomatous, endotheliomatous	comatose	died	PE
		38	F	L	trigone	IVH (SAH)	large	fibrous	drowsy	died	circulatory insufficiency with PE
Goran et al.	1965	55	M	R	lateral vent.	ICH	large	meningothelial with sarcomatous changes	stuporous	deficit	hemiplegia
Smith et al.	1975	14	F	L	trigone	IVH (SAH)	tumor: 2 x 3 cm	fibrous	somonolent	good recovery	
Hosaka et al.	1985	69	M	L	trigone	ICH/IVH	tumor: 5 x 4 x 3 cm	fibrous	stuporous	deficit	aphasia and disorientation
Lang et al.	1995	64	M	L	trigone	IVH/ tumor- surrounding	mass: 4 x 5 x 4 cm	fibrous	headache with LOC	deficit	hemianopsia and dysphasia
Murai et al.	1996	39	F	R	trigone	IVH/ tumor- surrounding	calcification: 2.5 cm	fibrous	headache with LOC	deficit	hemianopsia
Anekawa et al.	2001	61	M	R	trigone	SDH/SAH/ICH intratumor: (-)	mass: 5 cm	angiomatous	comatose	died	not operated
Lee et al.	2001	43	F	R	trigone	IVH intratumor: (+)	not so large	psammomatous	headache	good recovery	
Romeike et al.	2007	57	F	L	NS	ICH/IVH/ tumor-surrounding	large	fibrous	comatose	died	not operated
Fu et al.	2011	46	F	L	trigone	IVH/ tumor- surrounding	tumor: 2.9 x 2.7 cm	transitional	headache	deficit	hemianopsia
Moon et al.	2019	35	F	R	trigone	IVH/ tumor- surrounding	mass: 3.2 x 3.4 cm	meningothelial	drowsy	deficit	hemianopsia
Present case	2020	28	F	L	trigone	IVH/ICH	mass: 6.5 x 5 x 5 cm	fibrous	comatose	deficit	hemianopsia

*F* Female, *M* Male, *L* Left, *R* Right, *lateral vent.* Lateral ventricle, *NS* Not stated, *IVH* Intraventricular hemorrhage, *SAH* Subarachnoid hemorrhage, *ICH* Intracerebral hemorrhage, *SDH* Subdural hemorrhage, *LOC* Loss of consciousness, *PE* Pulumonary edema

The underlying mechanisms of spontaneous intratumoral hemorrhage in meningiomas are not fully understood, but have been well summarized in the review by Bosnjak et al., who also indicated that an increased bleeding tendency was found to be associated with two age groups (less than 30 y.o. and more than 70 y.o.), localized in the convexity (the most common site, with a 3-fold increased propensity) as well as intraventricular, and pathologically of the fibrous subtype [5]. They also described the events and issues contributing to the hemorrhage of meningioma as follows: (1) compensation enlargement of blood vessels with weakened walls in supplying or draining the meningioma, (2) angiomatous-like areas with thinned and friable vascular walls, (3) endothelial proliferation, subsequent vascular occlusion, and distal necrosis, (4) neovasculature in the granulation tissue around the necrosis, (5) neomembrane on the inner surface of the dura close to the meningioma, (6) tumor invasion of the vessel wall, and (7) other factors such as head trauma, anticoagulation treatment, or systemic disorders including hypertension or atherosclerosis [5]. Nevertheless, the exact cause of hemorrhage due to intraventricular meningiomas remains unclear, although several hypotheses have been put forward [10, 19]. Murai et al. suggested that intraventricular meningiomas tend to be clinically asymptomatic until they grow into large lesions. This may lead to an increase in neovascularization or the compression of tortuous vessels, giving rise to more bleeding [10]. Fu et al., in their case report and literature review, suggested that the rupture of abnormal developmental vessels within the meningioma would be the most likely source of hemorrhage [19]. Histological examinations of the 1st surgical specimen in our case revealed that massive hemorrhage and proliferative abnormal vessels were visible within the tumor. The origin of the hemorrhage in our case could thus, in part, have resulted from the rupture of neoplastic vessels within the tumor. However, it remains uncertain as to why not only intratumoral hemorrhage but also IVH and ICH occurred. Among the cases we collected from the literature, most patients also had ICH or ICH with a tumor-surrounding type of bleeding (Table 1). It is easy to infer that massive intratumoral hemorrhage could break through the surface of the tumor and cause IVH and ICH. In our case, proliferative abnormal vessels, such as softened thin-walled vessels, etc., and many closed vessels, suggesting previous bleeding, were also observed within the peri-tumor abundant collagen regions. Bleeding from abnormal vessels within such regions occasionally causes adhesion with the ventricular wall, and readily leads to IVH and/or ICH. We would therefore like to emphasize that peri-tumoral abundant collagen regions may contribute to IVH and ICH due to intraventricular meningioma at least in our case. On the other hand, as regard asymptomatic intratumoral bleeding, intraventricular meningiomas are more likely to be involved [3, 9, 10] (as mentioned above, previous bleeding was also suspected in our case), and this may cause the tumor to grow in volume and increase the additional opportunity for bleeding via multiple factors, eventually giving rise to massive bleeding. In fact, most patients we identified had very large lesions (long axis longer than 5 cm) at the onset of hemorrhage caused by intraventricular meningiomas, and these cases, including 2 cases that could not be operated on, had a poor prognosis. We would like to suggest therefore that surgical treatment for intraventricular meningioma should be undertaken even if the tumor is not large or even if the patient is asymptomatic when found. More recently, Mindermann et al. have reported 5 patients with ITM who underwent radiosurgery as the primary treatment, and concluded that the Gamma Knife or CyberKnife appears to be safe and effective for ITM [25]. Radiosurgery may thus also be an option for the treatment of ITM.

In conclusion, lateral ventricular meningioma presenting with intracranial hemorrhage such as acute stroke is a rare event, but recognition of such a pathogenesis is important. Although further accumulation of clinical data is needed to explore the cause of bleeding, we suggest that early surgery, including radiosurgery, should be undertaken in patients with lateral ventricular meningioma, even if it is not so large or asymptomatic.

## Data Availability

All data related to this case report are contained within the manuscript.

## Abbreviations

CT: Computed tomography
ICH: Intracerebral hemorrhage
ITM: Intraventricular trigonal meningioma
IVH: Intraventricular hemorrhage
MRI: Magnetic resonance imaging
